# Prospective study comparing olfactory function in patients undergoing nasoseptal and rescue flap

**DOI:** 10.1016/j.bjorl.2026.101931

**Published:** 2026-10-15

**Authors:** Ricardo Landini Lutaif Dolci, Leticia Sayuri Yada, Leandro Meira Martins, Dino Rafael Perez, Matheus Cunha Ferreira de Castro Tolentino, Américo Rubens Leite dos Santos, Paulo Roberto Lazarini

**Affiliations:** a Santa Casa de Misericórdia de São Paulo, Department of Otorhinolaryngology, São Paulo, SP, Brazil; b Faculdade de Ciências Médicas da Santa Casa de Misericórdia de São Paulo, São Paulo, SP, Brazil; c Santa Casa de Misericórdia de São Paulo, Department of Neurosurgery, São Paulo, SP, Brazil

**Keywords:** Olfaction, Skull base, Endoscopic, Flap

## Abstract

**Objective:**

Endoscopic skull base surgery, whether for treatment or reconstruction, can affect olfaction. Assessing whether the rescue flap technique leads to better olfactory recovery than the nasoseptal flap is essential for developing criteria to guide surgical flap selection. The primary objective of this study was to assess olfactory function in patients undergoing endoscopic skull base surgery with either a nasoseptal or rescue flap. The secondary objective was to evaluate nasal symptoms.

**Methods:**

Olfactory and nasal symptom assessments were performed using the Connecticut Chemosensory Clinical Research Center (CCCRC) olfactory test and the Sino-nasal Outcome Test (SNOT-22) questionnaire at four time points: 24 h preoperatively, and at 1, 3, and 6 months postoperatively.

**Results:**

A total of 47 patients were included, divided into two groups: nasoseptal flap (n = 28) and rescue flap (n = 19). Both groups demonstrated preserved olfactory function and site-specific quality-of-life scores when comparing preoperative and 6-month postoperative data.

**Conclusion:**

The results indicate that there was no significant change in olfactory function between groups over the evaluated time points, and no long-term deterioration in olfactory function or site-specific quality of life. Neither group demonstrated an earlier olfactory recovery that was statistically significant. Additionally, the SNOT-22 subdomains related to sleep disturbances and psychological symptoms showed progressive improvement, suggesting enhanced sleep quality and improved mental well-being.

**Level of evidence:**

2.

## Highlights

• Prospective comparison of nasoseptal vs. rescue flap.
• Olfaction recovers to baseline by 6-months in both groups.
• SNOT-22 improves progressively postoperatively.
• Sleep and psychological domains show major gains.

## Introduction

Endoscopic skull base surgery has become the gold standard for the treatment of skull base tumors. However, because the endonasal approach requires manipulation of the nasal cavity and may involve the olfactory epithelium, it has been associated with postoperative olfactory impairment.^1^

A technical aspect of endoscopic skull base surgery that remains underexplored in the literature is the relationship between flap type and postoperative olfactory function quantification.^2^, ^3^, ^4^

In 2006, Hadad et al. described the use of the nasoseptal flap with the objective of reducing cerebrospinal fluid (CSF) leak rates in endonasal procedures used to access the skull base, utilizing a pedicled flap based on the posterior nasoseptal artery (a posterior branch of the sphenopalatine artery).^5^ In 2011, Rivera-Serrano et al. published a study on the rescue flap for procedures in which CSF leakage would not be initially expected, but in which conversion into a nasoseptal flap could be performed if necessary. Unlike the nasoseptal flap, the rescue flap requires less manipulation of the nasal septal mucosa, resulting in reduced trauma to the olfactory epithelium.^6^

Although previous studies have compared respiratory function and the impact on olfaction across various flap techniques, this study aimed to compare subsequent olfactory function and quality of life in patients who underwent skull base surgery using either the nasoseptal flap or the rescue flap.

## Methods

This is a prospective study involving patients who underwent endoscopic skull base surgery for tumor removal or cerebrospinal fluid (CSF) leak closure. All patients were interviewed and completed the SNOT-22 questionnaire 24-hs preoperatively and at 1-, 3-, and 6-months afterward. Additionally, the CCCRC test was applied at the same time points to evaluate qualitatively and quantitatively olfactory function.^7^^,^^8^

The study was conducted at the Department of Otorhinolaryngology of Santa Casa de São Paulo. All participants provided written informed consent. Data collection took place between November 2020 and July 2024.

### Olfactory test

Olfactory function was assessed using the CCCRC test (medsmell@, São Paulo, Brazil), administered 24 h before surgery and at 1-, 3-, and 6-months postoperatively. The CCCRC test comprises two parts: threshold and identification tests. The threshold test involved presenting increasing 1-butanol concentrations to identify the lowest detectable level. The concentrations used were 0.00061%, 0.0018%, 0.0055%, 0.016%, 0.049%, 0.15%, 0.44%, 1.3%, and 4% in deionized nanopure water. Solutions were stored in containers and presented alongside a control bottle with water. Patients occluded one nostril and chose which had the stronger odor. If incorrect, a stronger concentration was presented, and the test repeated. Four correct answers defined the threshold. The process was then repeated for the other nostril. The identification test used opaque containers with talcum powder, cocoa powder, cinnamon, ground coffee, naphthalene, paçoca (Brazilian peanut candy), and soap. Again, the patient occluded one nostril and identified the smell. The process was repeated with the other nostril. If an error occurred, the patient could retry the same item; if correct, the mistake was disregarded. Final scores were the average of threshold and identification results from both nostrils, categorized as: 0–1.75 = anosmia; 2–3.75 = severe hyposmia; 4–4.75 = moderate; 5–5.75 = mild; 6 = normosmia.

### Questionnaire

The SNOT-22 questionnaire is the most widely used instrument for assessing site-specific sinonasal Quality of life (QoL).^7^, ^8^, ^9^ It consists of 22 questions, each scored on a scale from 0 to 5. The total score provides a numerical representation of the patient's QoL, with higher scores indicating poorer sinonasal-related quality of life.

### Surgical technique

The surgical approach was standardized in all cases to ensure procedural uniformity and minimize anatomical variables that could interfere with olfactory assessment. In all patients, the procedure consisted of a right anterior and posterior ethmoidectomy associated with resection of the right middle turbinate, while the contralateral anterior and posterior ethmoid and middle turbinate were preserved to maintain the integrity of the olfactory structures on the non-operated side.

Surgical techniques were then refined according to the tumor’s location, size, and underlying pathology to ensure the most appropriate approach for each individual case. Following determination of the surgical plan, patients were allocated into two groups: nasoseptal flap and rescue flap. The nasoseptal flap was employed in cases where an intraoperative Cerebrospinal Fluid (CSF) leak occurred, whereas the rescue flap was indicated for smaller lesions without intraoperative CSF leak, with closure performed using a graft from the middle turbinate mucosa.

### Inclusion and exclusion criteria

The inclusion criteria were patients who underwent endoscopic transnasal surgery for tumor resection in the skull base region and who completed the SNOT-22 questionnaire and the CCCRC test at 24 -hs preoperatively, and at 1-, 3-, and 6-months postoperatively.

Exclusion criteria included: patients unable to complete the CCCRC test and the SNOT-22 questionnaire; presence of preoperative anosmia; patients who underwent combined surgical approaches (i.e., endoscopic transsphenoidal approach plus craniotomy); and patients who underwent transcribriform surgical approaches.

### Data analysis

All data were compiled in spreadsheets using RedCapR for subsequent statistical analysis. Unpaired tests analyzed the parameters. A significance level of p < 0.05 was adopted. Statistical software included SPSS v26 (2019), Minitab 21.2 (2022), and Excel 2010. The Mann–Whitney *U*-test was used to compare demographic and anthropometric data between groups, as well as to compare the change in CCCRC score between the preoperative and 6-month postoperative evaluations (ΔCCCRC) in patients who underwent septoplasty and those who did not.

For intra-group comparisons across timepoints for CCCRC and SNOT-22, the Friedman and Wilcoxon signed-rank tests were used. For intergroup temporal comparisons, the Mann–Whitney test was also applied. These comparisons were performed at each timepoint and for delta (Δ) values between them. The delta was defined as the simple arithmetic difference between two timepoints, as follows: Δ1 = 1-month – Preoperative; Δ2 = 3-months – Preoperative; Δ3 = 6-months – Preoperative; Δ4 = 3-months – 1-month; Δ5 = 6-months – 1-month; Δ6 = 6-months – 3-months.

A negative delta value indicates a reduction between timepoints, whereas a positive value reflects an increase. Additionally, for intragroup temporal comparisons of SNOT-22 domains, the Wilcoxon signed-rank test was used.

## Results

### Intergroup comparison of demographic and anthropometric factors

A total of 71 patients were initially evaluated in this study. After applying the eligibility criteria, 24 patients were excluded, resulting in a final sample of 47 patients. The nasoseptal flap group included 28 patients with a mean age of 43.5-years (SD = 14.8), and a median age of 43.5. The rescue flap group consisted of 19 patients, with a mean age of 56.4-years (SD = 15) and a median age of 57.

### Intragroup comparison of CCCRC and Snot-22 scores across timepoints

To assess outcome progression ‒ comparison across timepoints within each group for CCCRC and SNOT-22 ‒ the Friedman and Wilcoxon tests were used.

In the nasoseptal flap group, CCCRC scores showed a statistically significant improvement from preoperative to postoperative, especially at 6-months. In the rescue flap group, a similar trend was seen, except between preoperative and 6-month scores, which showed no statistical difference (Table 1, Fig. 1).

**Table 1:** Intragroup temporal comparison of p-values for CCCRC scores.

		Preoperative	1-month	3-months
Nasoseptal Flap	1m	<0.001		
	3m	0.009	<0.001	
	6m	0.017	<0.001	<0.001
Rescue Flap	1m	<0.001		
	3m	0.019	0.001	
	6m	0.811	0.003	0.035

**Fig. 1:**
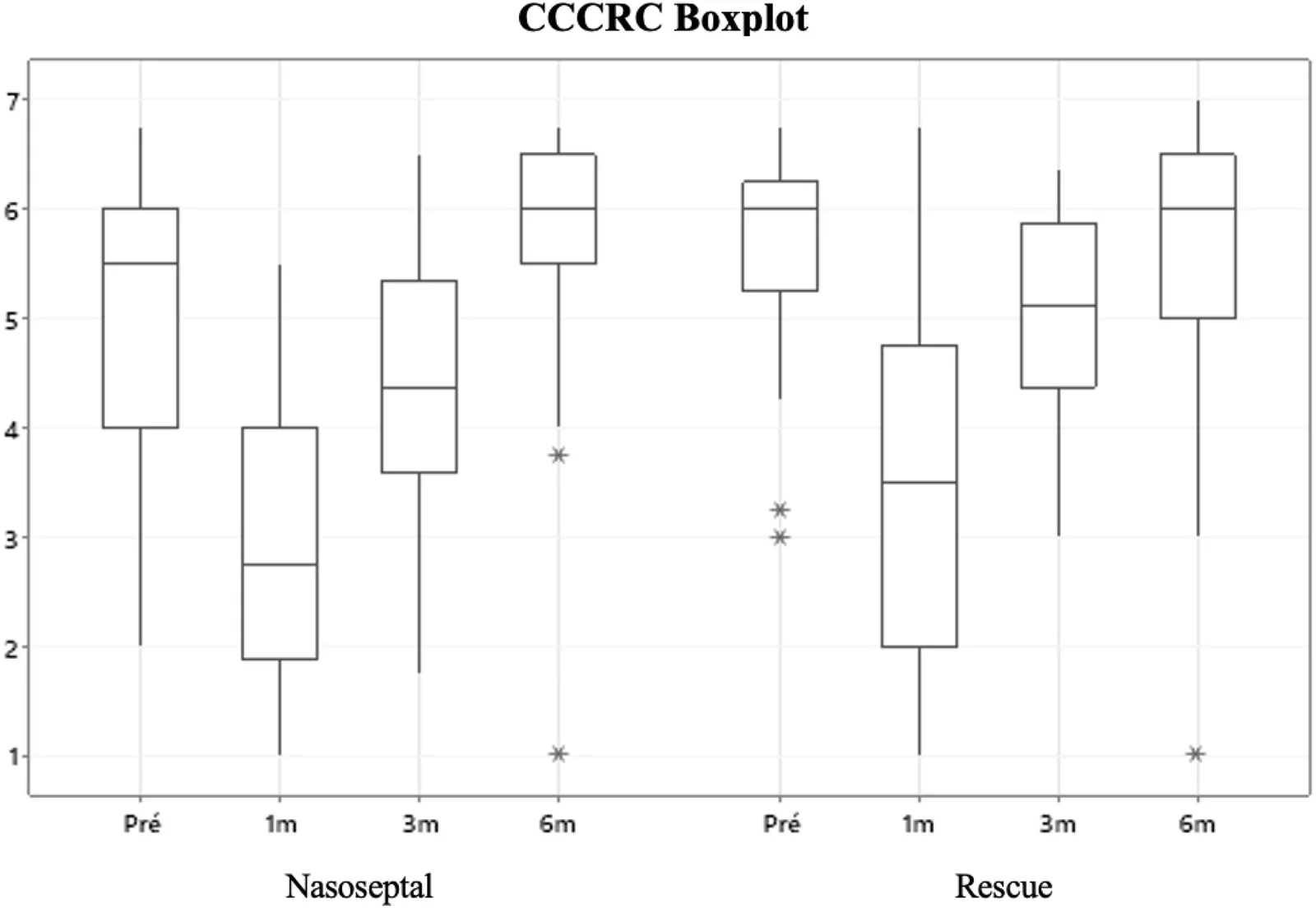
Evolution of CCCRC scores by flap type over time.

For SNOT-22, neither group showed significant differences between pre-op and 1-month, or between 3- and 6-month scores. Other comparisons showed significant symptom improvement over time (Table 2, Fig. 2).

**Table 2:** Intragroup temporal comparison of p-values for SNOT-22 scores.

		Preoperative	1-month	3-months
Nasoseptal	1m	0.223		
	3m	0.002	<0.001	
	6m	0.001	<0.001	0.754
Rescue	1m	0.334		
	3m	0.012	0.004	
	6m	0.003	<0.001	0.078

**Fig. 2:**
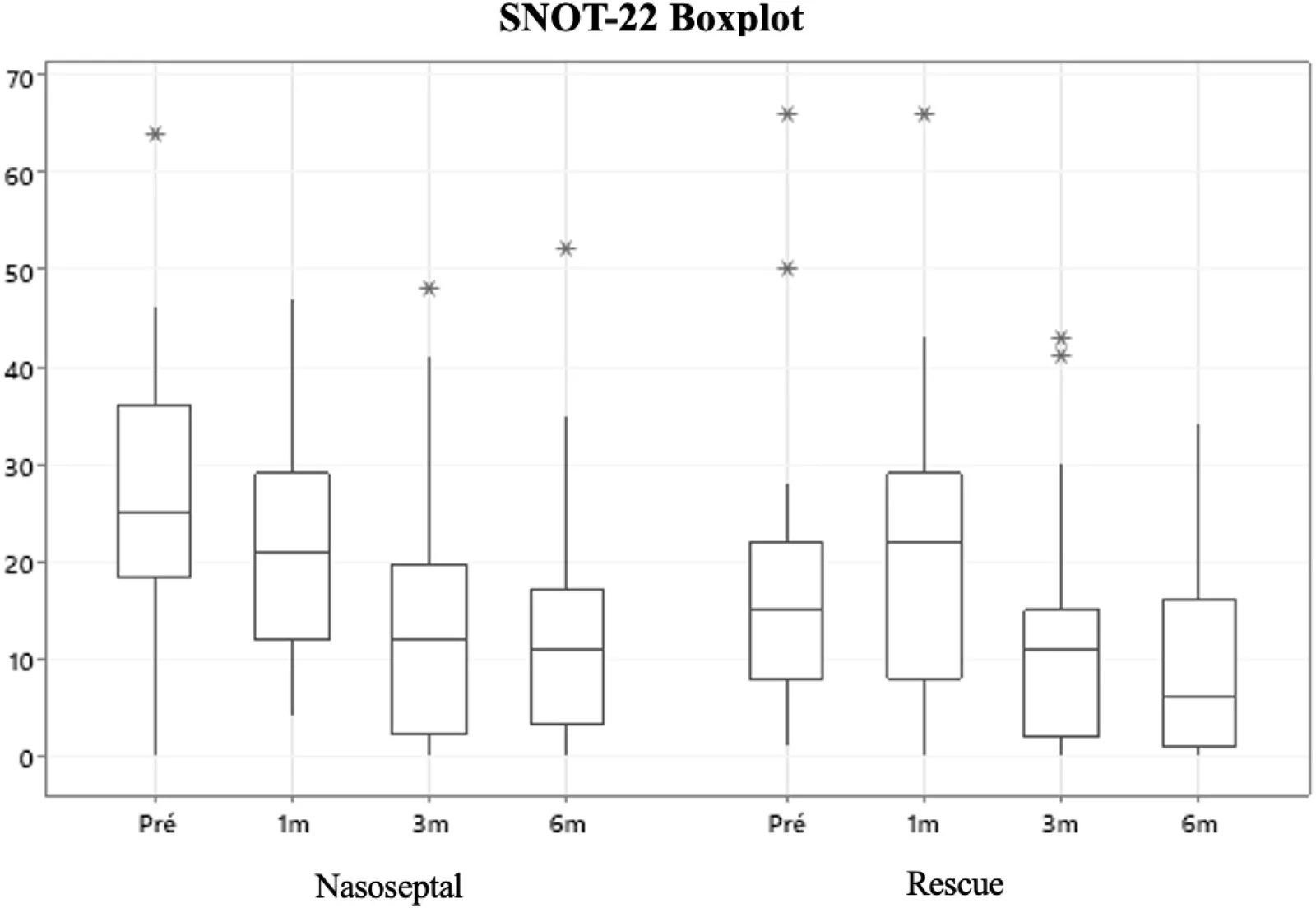
Temporal progression of SNOT-22 scores according to flap type.

### Intragroup temporal comparison for Snot-22 domains

Delta analysis revealed statistically significant differences in the psychological dysfunction and sleep dysfunction domains, with score reductions observed in both groups. In the psychological dysfunction domain, the mean score in the nasoseptal group decreased from 11.07 to 3.89 (p *<* 0.001), while in the rescue group, it decreased from 8.21 to 2.68 (p *=* 0.002).

In the sleep dysfunction domain, the mean score in the nasoseptal group dropped from 8.75 to 3.82 (p = 0.001), and in the rescue group from 5.74 to 2.32 (p *=* 0.006).

Additionally, delta analysis of the rhinologic domain in the rescue group revealed a statistically significant difference, with mean scores increasing from 2.37 preoperatively to 3.37 at 6-months (p = 0.014; Table 3).

**Table 3:** Intragroup temporal comparison of SNOT-22 domains.

Group			Mean	Median	SD	Q1	Q3	n	CI	p-value
Rhinologic	Nasoseptal	Pre	4.43	3	4.98	0	8	28	1.84	0.97
		6m	3.96	3	4.24	0	6.25	28	1.57	
	Rescue	Pre	2.37	1	3.58	0	3.5	19	1.61	0.014
		6m	3.37	3	3.11	1.5	5	19	1.4	
Extra-nasal Rhinologic	Nasoseptal	Pre	2.96	1.5	3.44	0	4.25	28	1.27	0.512
		6m	2.25	1	2.62	0	4	28	0.97	
	Rescue	Pre	2	2	1.86	0	3.5	19	0.83	0.569
		6m	1.58	2	1.61	0	2	19	0.72	
Auditory/ Facial	Nasoseptal	Pre	3.75	3	3.36	1	6	28	1.25	0.16
		6m	2.46	1	3.5	0	4.25	28	1.3	
	Rescue	Pre	3.84	2	3.96	0	7	19	1.78	0.225
		6m	2.68	2	2.87	0	4.5	19	1.29	
Psychological Dysfunction	Nasoseptal	Pre	11.07	12	6.13	8.25	14.3	28	2.27	<0.001
		6m	3.89	3	4.77	0	6	28	1.77	
	Rescue	Pre	8.21	6	8.23	2.5	10	19	3.7	0.002
		6m	2.68	0	4.7	0	3	19	2.11	
Sleep Dysfunction	Nasoseptal	Pre	8.75	9	5.76	3.75	12	28	2.13	0.001
		6m	3.82	3	4.39	0	6.25	28	1.63	
	Rescue	Pre	5.74	4	5.89	1.5	7.5	19	2.65	0.006
		6m	2.32	0	4,16	0	2	19	1.87	

### Intergroup comparison

#### Temporal intergroup comparison for CCCRC

When evaluating the groups across timepoints (preoperative, 1-month, 3-months, and 6-months postoperatively), no statistically differences were were found within or between timepoints (Table 4).

**Table 4:** Temporal intergroup comparison for CCCRC scores.

Group		Mean	Median	SD	Q1	Q3	N	CI	p-value
Pre	Nasoseptal	5.11	5.50	1.35	4.50	6.00	28	0.50	0.239
	Rescue	5.57	6.00	1.04	5.25	6.25	19	0.47	
1m	Nasoseptal	3.04	2.75	1.37	2.13	4.00	28	0.51	0.344
	Rescue	3.57	3.50	1.79	2.13	4.75	19	0.80	
3m	Nasoseptal	4.43	4.38	1.24	3.78	5.28	28	0.46	0.106
	Rescue	5.02	5.13	0.95	4.56	5.81	19	0.43	
6m	Nasoseptal	5.57	6.00	1.26	5.50	6.50	28	0.47	0.878
	Rescue	5.50	6.00	1.55	5.25	6.38	19	0.69	
Delta 1	Nasoseptal	−2.06	−2.00	1.65	−3.00	−0.88	28	0.61	0.905
	Rescue	−2.00	−2.00	1.48	−3.13	−0.88	19	0.67	
Delta 2	Nasoseptal	−0.68	−0.81	1.21	−1.31	0.19	28	0.45	0.948
	Rescue	−0.55	−0.63	0.97	−1.13	−0.06	19	0.43	
Delta 3	Nasoseptal	0.46	0.38	0.90	0.00	1.00	28	0.34	0.117
	Rescue	−0.07	0.00	1.04	−0.25	0.63	19	0.47	
Delta 4	Nasoseptal	1.38	1.13	1.45	0.22	2.16	28	0.54	0.688
	Rescue	1.45	1.25	1.33	0.69	2.44	19	0.60	
Delta 5	Nasoseptal	2.53	2.25	1.55	1.50	3.81	28	0.57	0.328
	Rescue	1.93	1.75	2.02	1.00	3.50	19	0.91	
Delta 6	Nasoseptal	1.14	1.31	1.04	0.09	2.00	28	0.38	0.099
	Rescue	0.48	0.75	1.44	0.31	1.19	19	0.65	

#### Temporal intergroup comparison for Snot-22

When analyzing the timepoints and the deltas between each period, the only statistically significant difference was observed in the preoperative SNOT-22 scores, with a mean of 27.00 in the Nasoseptal group and 18.79 in the Rescue group (p *=* 0.019; Table 5). No patient in either group had chronic rhinosinusitis or nasal polyps on preoperative evaluation.

**Table 5:** Temporal intergroup comparison for SNOT-22 scores.

Group		Mean	Median	SD	Q1	Q3	N	CI	p-value
Pre	Nasoseptal	27.00	25.0	14.65	18.8	36.0	28	5.43	0.019
	Rescue	18.79	15.0	15.77	9.5	22.0	19	7.09	
1m	Nasoseptal	22.21	21.0	11.11	14.0	29.0	28	4.11	0.587
	Rescue	21.53	22.0	16.45	8.0	29.0	19	7.40	
3m	Nasoseptal	14.11	12.0	13.28	2.8	17.3	28	4.92	0.687
	Rescue	12.74	11.0	12.87	3.0	15.0	19	5.79	
6m	Nasoseptal	13.04	11.0	12.74	3.8	17.0	28	4.72	0.322
	Rescue	9.95	6.0	11.09	1.5	12.5	19	4.99	
Delta 1	Nasoseptal	−4.79	−1.5	17.19	−15.3	6.5	28	6.37	0.143
	Rescue	2.74	3.0	13.71	−6.5	15.0	19	6.17	
Delta 2	Nasoseptal	−12.89	−10.5	19.20	−23.3	−0.5	28	7.11	0.212
	Rescue	−6.05	−2.0	9.69	−12.5	−0.5	19	4.36	
Delta 3	Nasoseptal	−13.96	−12.5	18.52	−22.0	−0.8	28	6.86	0.454
	Rescue	−8.84	−8.0	10.45	−15.5	−2.0	19	4.70	
Delta 4	Nasoseptal	−8.11	−9.0	8.09	−12.0	−3.8	28	3.00	0.991
	Rescue	−8.79	−8.0	11.34	−16.5	0.0	19	5.10	
Delta 5	Nasoseptal	−9.18	−11.0	10.17	−13.3	−7.0	28	3.77	0.745
	Rescue	−11.58	−9.0	8.45	−17.0	−6.0	19	3.80	
Delta 6	Nasoseptal	−1.07	0.0	8.41	−4.0	2.5	28	3.12	0.260
	Rescue	−2.79	−1.0	8.24	−6.5	0.0	19	3.71	

#### Temporal intergroup comparison for Snot-22 domains

Preoperatively, significant differences were found in Psychological Dysfunction and Sleep Dysfunction domains. In the Psychological Dysfunction domain, the mean score in the nasoseptal group was 11.07 (SD = 6.13), compared to 8.21 (SD = 8.23) in the rescue group, with a p-value of 0.04. In the Sleep Dysfunction domain, the mean score was 8.75 (SD = 5.76) in the nasoseptal group and 5.74 (SD = 5.89) in the rescue group, with a p-value of 0.048.

No statistically significant differences were identified between groups in the remaining timepoints or in the deltas between timepoints (Table 6).

**Table 6:** Temporal intergroup comparison for SNOT-22 domains.

Group			Mean	Median	SD	Q1	Q3	N	CI	p-value
Rhinologic	Pre	Nasoseptal	4.43	3	4.98	0	8	28	1.84	0.169
		Rescue	2.37	1	3.58	0	3.5	19	1.61	
	6m	Nasoseptal	3.96	3	4.24	0	6.25	28	1.57	0.904
		Rescue	3.37	3	3.11	1.5	5	19	1.4	
	Delta	Nasoseptal	−0.46	0	5.41	−2	3	28	2	0.281
		Rescue	1	0	3.21	0	2.5	19	1.45	
Extra-nasal Rhinologic	Pre	Nasoseptal	2.96	1.5	3.44	0	4.25	28	1.27	0.642
		Rescue	2	2	1.86	0	3.5	19	0.83	
	6m	Nasoseptal	2.25	1	2.62	0	4	28	0.97	0.648
		Rescue	1.58	2	1.61	0	2	19	0.72	
	Delta	Nasoseptal	−0.71	0	4.59	−3.25	2	28	1.7	0.939
		Rescue	−0.42	0	2.43	−1.5	1.5	19	1.09	
Auditory/ Facial	Pre	Nasoseptal	3.75	3	3.36	1	6	28	1.25	0.785
		Rescue	3.84	2	3.96	0	7	19	1.78	
	6m	Nasoseptal	2.46	1	3.5	0	4.25	28	1.3	0.458
		Rescue	2.68	2	2.87	0	4.5	19	1.29	
	Delta	Nasoseptal	−1.29	−1	4.88	−4	1.25	28	1.81	0.931
		Rescue	−1.16	−1	4.1	−4.5	1	19	1.84	
Psychological Dysfunction	Pre	Nasoseptal	11.07	12	6.13	8.25	14.25	28	2.27	0.04
		Rescue	8.21	6	8.23	2.5	10	19	3.7	
	6m	Nasoseptal	3.89	3	4.77	0	6	28	1.77	0.252
		Rescue	2.68	0	4.7	0	3	19	2.11	
	Delta	Nasoseptal	−7.18	−8.5	6.49	−12	−3	28	2.4	0.453
		Rescue	−5.53	−5	5.74	−10	−1	19	2.58	
Sleep Dysfunction	Pre	Nasoseptal	8.75	9	5.76	3.75	12	28	2.13	0.048
		Rescue	5.74	4	5.89	1.5	7.5	19	2.65	
	6m	Nasoseptal	3.82	3	4.39	0	6.25	28	1.63	0.119
		Rescue	2.32	0	4.16	0	2	19	1.87	
	Delta	Nasoseptal	−4.93	−3	6.77	−8.25	0	28	2.51	0.752
		Rescue	−3.42	−3	4.39	−6.5	0	19	1.97	

### Power analysis

Using the G*Power 3.1.9.4 software to calculate the statistical power of this study, we specified an effect size of 0.53, based on the difference between the Nasoseptal group (x¯ = 27.00; σ = 14.65) and the Rescue group (x¯ = 18.79; σ = 15.77) for preoperative SNOT-22 scores. According to Cohen’s classification, this effect size is considered medium (small = 0.2 and large = 0.8).

Considering a significance level of 0.05 and a total sample size of 47 subjects (28 in the Nasoseptal group and 19 in the Rescue group), the achieved power of this sample was 0.816 (81.6%).

### Association between septoplasty and postoperative olfactory function

To investigate whether septoplasty contributed to postoperative olfactory improvement, the change in CCCRC score from baseline to the 6-month evaluation was compared between patients who underwent septoplasty and those who did not. No statistically significant difference was observed between the groups (median ΔCCCRC = 0.0 [IQR -0.25 to 0.50] vs. 0.5 [IQR 0.0–1.0], Mann–Whitney *U* = 366, p = 0.056).

## Discussion

Current studies on olfactory outcomes following endoscopic skull base surgery suggest that there is no significant long-term postoperative impact. Although transient olfactory alterations may occur in the early postoperative period, most patients tend to return to baseline levels.^10^^,^^11^ In the systematic review by Yin et al. (2019), only one study ‒ conducted by Dolci et al. (2017) ‒ was identified that evaluated the relationship between the use of the nasoseptal flap and olfactory function using the CCCRC test. In that study, only 10 out of 41 patients presented postoperative hyposmia or anosmia. However, to date, no studies have directly compared patients undergoing nasoseptal flap versus rescue flap reconstruction to assess whether differences exist in the early postoperative recovery of olfactory function depending on the type of flap used.^12^^,^^13^

However, the present study aimed to evaluate whether olfactory function in patients undergoing endoscopic skull base surgery returns to their preoperative baseline and whether this recovery process is influenced by the type of flap employed. Specifically, the study compared the nasoseptal flap with the rescue flap, considering that the former involves more extensive manipulation of the nasal cavity. The objective was to determine whether one particular flap type may facilitate a faster or more effective recovery of olfactory function.

Our initial hypothesis was that the rescue flap, due to its reduced manipulation of nasal structures, might facilitate a faster recovery of olfactory function compared to the nasoseptal flap ‒ particularly during the early postoperative period between one and three months. However, the findings of this study did not reveal any significant difference in olfactory outcomes between the two groups. Preoperative olfactory scores were comparable, with means of 5.11 and 5.57 in the nasoseptal and rescue groups, respectively (p *=* 0.239), and this similarity persisted across all postoperative assessments, despite minor variations in mean scores.

Both groups demonstrated a return to baseline olfactory function by six months after surgery. As expected, olfactory performance declined significantly at the one-month mark, followed by progressive improvement, ultimately reaching preoperative levels ‒ a trend consistent with the observations reported by Carvalho et al.^14^ This outcome, however, diverges from the findings of Tam et al.,^15^ who reported a negative impact on olfactory function associated with the use of the nasoseptal flap. The authors suggest that this finding may be due to the fact that a high, horizontal superior incision was performed, preserving the olfactory epithelium at a distance of less than 1 cm from the skull base. In contrast, in the present study, when creating the nasoseptal flap an effort was made to preserve the olfactory epithelium by placing the superior incision at a minimum distance of 1.5 cm from the upper third of the septal mucosa.

Although a recovery of olfactory function to preoperative levels was observed in most cases, in some there was an improvement beyond baseline values, a finding also reported by Griffins et al.^16^ While surgical correction of septal deviations may theoretically improve nasal airflow and olfactory function, our additional analysis found no significant association between septoplasty and postoperative olfactory improvement. Therefore, the improvement beyond baseline observed in our cohort is likely multifactorial and may also reflect postoperative healing and adherence to nasal hygiene practices, particularly regular saline nasal irrigation (0.9% NaCl).^17^^,^^18^

The SNOT-22 test is a modified version of the SNOT-20 questionnaire, originally developed by Piccirillo et al.,^19^ and later expanded by Hopkins et al.^20^ to include 22 items assessing symptoms related to the nasal cavity context. Although it has not yet been formally validated for patients undergoing Endoscopic Skull Base Surgery (ESBS) and does not encompass all possible associated symptoms, it is widely used in the postoperative evaluation of transnasal endoscopic skull base procedures.^21^, ^22^, ^23^ This is due to its simplicity ‒ consisting of 22 questions covering nasal, extra-nasal, and auditory/facial symptoms, as well as sleep and psychological dysfunction ‒ and the use of an easily understandable scoring scale. Moreover, the anatomical regions addressed intraoperatively in ESBS are similar to those involved in Functional Endoscopic Sinus Surgery (FESS), supporting the tool’s applicability in this context.

In the SNOT-22 assessment, patients in both the nasoseptal and rescue flap groups showed a progressive reduction in questionnaire scores over time. The most symptomatic period occurred within the first 30 postoperative days, likely due to increased crust formation, secretions, and mucosal edema. However, both groups demonstrated significant progressive improvement in scores during subsequent evaluations, ultimately achieving values lower than the preoperative baseline, with statistically significant differences.

When analyzing the SNOT-22 subdomains, statistically significant improvements were observed in the psychological dysfunction and sleep dysfunction domains, indicating substantial postoperative benefits in these areas. In the psychological domain, both groups exhibited a marked decrease in scores, suggesting that the surgery positively impacted patients’ mental health, potentially reducing symptoms such as anxiety and emotional distress associated with skull base tumors. Furthermore, a significant improvement in the sleep dysfunction domain was also observed in both groups. This suggests that, postoperatively, patients experienced improved sleep quality ‒ likely related to reduced nasal obstruction and improved breathing, as well as the emotional factors ‒such as frustration, restlessness, irritability, sadness, and shame ‒ that influence sleep patterns. Additionally, patients in the rescue flap group showed improvement in the rhinologic domain. These findings are consistent with the study by Dolci et al.,^24^ which reported a significant improvement in the site-specific SNOT-22 questionnaire among patients undergoing endoscopic transsphenoidal surgery for resection of pituitary adenomas using the nasoseptal flap.

One limitation of this study is the heterogeneity of the sample, as patients undergoing skull base surgery presented with different underlying pathologies and required distinct surgical approaches for tumor removal. To mitigate this bias, a standardized nasosinusal surgical approach was employed. This included the removal of the anterior and posterior ethmoidal cells on the right side, along with a wide sphenoidectomy. The superior boundary was defined by the transition between the cribriform plate and the planum sphenoidale, while the lateral boundary was the lateral wall of the sphenoid sinus. Additionally, a partial unilateral resection of the middle turbinate was performed in all patients. In three cases, a transpterygoid approach was used, involving maxillary sinus opening, which is not known to interfere with olfactory function.

It is important to highlight that this study represents only an initial step toward understanding the impact that different flaps may have on olfactory function and quality of life in patients undergoing endoscopic skull base surgery. The results presented here, derived from a prospective and innovative study, contrast with the existing literature, which is largely based on retrospective studies. Moreover, the evaluation of SNOT-22 subdomains ‒ still scarcely explored in the literature ‒ adds to the novelty of the findings. These results support the notion that choosing a more robust flap, such as the nasoseptal flap, can be done with confidence, as this approach does not appear to negatively impact quality of life, either in terms of olfaction or other site-specific functions.

Furthermore, the study’s statistical power of 0.816 (81.6%) demonstrated that the sample size was adequate to detect significant differences between the groups analyzed. This result reinforces the statistical robustness of the analysis and suggests that the number of participants was sufficient to minimize the risk of Type II error, thereby increasing the reliability of the conclusions obtained.

Thus, flap selection should primarily be guided by the need for adequate skull base defect closure, given the absence of statistically significant differences in olfactory function and quality of life between patients treated with nasoseptal and rescue flaps.

## Conclusion

The results demonstrated that there was no deterioration in olfactory function and no differences in quality-of-life scores between the groups analyzed in the long term, nor across the evaluated time points. This consistency remained even when considering that the rescue flap involves less nasal morbidity during the intraoperative period. Furthermore, the SNOT-22 subdomains related to sleep disturbances and psychological symptoms showed progressive improvement over time, suggesting a positive impact on patients' mental health.

## ORCID ID

Ricardo Landini Lutaif Dolci: 0000-0002-8404-8495

Leticia Sayuri Yada: 0009-0005-4381-9053

Leandro Meira Martins: 0000-0003-1967-8236

Dino Rafael Perez: 0000-0001-5167-0029

Matheus Cunha Ferreira de Castro Tolentino: 0009-0003-9116-0519

Américo Rubens Leite dos Santos: 0000-0003-4213-2709

Paulo Roberto Lazarini: 0000-0002-4860-8088

## Authors’ contributions

Paulo R. Lazarini: Writing, analysis and design. Ricardo L. L. Dolci: Writing, analysis and design. Leticia S. Yada: Data collection, writing, analysis and design. Leandro Meira Martins: Data collection. Dino Rafael Perez: Writing. Matheus Cunha Ferreira de Castro Tolentino: Writing. Américo Rubens Leite dos Santos: Writing.

## Consent to participate

Informed consent was obtained from legal guardians.

## Consent to publish

Informed consent was obtained from legal guardians.

## Ethics approval

CAAE: 40011920.8.0000.5479.

## Data

Not applicable.

## Materials and/or Code availability

Not applicable.

## Funding

The authors declare that they have no relevant financial or material interests related to the research described in this article.

## Data availability statement

The authors declare that all data are available in repository.

## Declaration of competing interest

The authors have no competing interests to declare that are relevant to the content of this article.
